# A conjoint analysis study on self-sampling for human papillomavirus (HPV) testing characteristics among black women in Indiana

**DOI:** 10.1186/s12905-020-00921-x

**Published:** 2020-03-19

**Authors:** Erika Biederman, Victoria Champion, Gregory Zimet

**Affiliations:** 1 Indiana University School of Nursing, 600 Barnhill Drive, NU317, Indianapolis, IN 46202 USA; 2 Department of Pediatrics–Adolescent Medicine, Indiana University School of Medicine, 410 West 10th Street, Suite 1001, Indianapolis, IN 46202 USA

**Keywords:** HPV testing, Self-sampling, Conjoint analysis

## Abstract

**Background:**

Self-sampling for HPV testing may be a method to increase overall cervical cancer screening rates among Black women, who are underscreened for cervical cancer in parts of the US. The purpose of this study was to assess preferred characteristics for delivery of HPV self-sampling kits, return of HPV self-sampling kits, and communication of HPV test results and explore sociodemographic factors (income, education, and marital status) associated with acceptability of self-sampling for HPV testing.

**Methods:**

Survey data were gathered at an Indiana minority health fair. Participants evaluated 9 scenarios that varied along 3 dimensions: HPV self-sampling kit delivery (mail, pharmacy pick-up, or clinic pick-up), HPV self-sampling kit return (mail, pharmacy drop-off, or clinic drop-off), and HPV test results (mail, phone call, or text message). The 9 scenarios were produced from a fractional factorial design and rated on a 0 to 100 scale. Ratings-based conjoint analysis (RBCA) determined how each dimension influenced ratings. A measure for acceptability of self-sampling was obtained from the ratings of all 9 scenarios. The acceptability measure was regressed on sociodemographics.

**Results:**

The 98 participants ranged in age from 21 to 65 (M = 45). Across the 9 scenarios, overall acceptability to self-sample had a mean of 60.9 (SD = 31.3). RBCA indicated that HPV self-sampling kit return had the most influence on ratings, followed by HPV self-sampling kit delivery, and finally, HPV test result communication. Thirty-six percent of participants rated all self-sampling scenarios the same. Sociodemographic characteristics were not associated with acceptability of self-sampling.

**Conclusions:**

Self-sampling for HPV testing was found to be generally acceptable to Black women in this pilot survey study. This information could be used by researchers developing self-sampling interventions and the implementation of self-sampling among providers.

## Background

Black women in the United States (US) continue to bear an unequal burden of cervical cancer, which is a salient problem in Indiana where Black women have a 23% higher cervical cancer incidence [1] and 25% higher mortality [2] rate compared to White women. Additionally, Black women in Indiana have one of the lowest cervical cancer screening rates in the nation with over 25% of Black women outside of screening guidelines [3]. Currently, the United States Preventive Services Task Force (USPSTF) guidelines for cervical cancer screening include: clinic-based screening options with 1.) cytology alone every 3 years (age 21–65), 2) co-testing of cytology and human papillomavirus (HPV) every 5 years (age 30–65), or 3) an HPV test alone every 5 years (age 30–65) [4]. Self-sampling for human papillomavirus (HPV) testing, a method for women to collect their own cervicovaginal sample, is one technology that has been explored in the US and internationally as an alternative to clinic-based cervical cancer screening. Self-sampling for HPV testing is well documented as having comparable sensitivity to clinician-collected HPV samples [5, 6]. Self-sampling for HPV testing may be a method to overcome the many structural and personal barriers among Black women related to clinic-based testing including transportation [7, 8], childcare [7, 8], embarrassment [9], pain [10], and fear [8, 10]. Self-sampling for HPV testing could increase cervical cancer screening rates and decrease morbidity and mortality from cervical cancer.

Self-sampling for HPV testing has been integrated into screening programs in other countries (the Netherlands [11] and Australia [12]) and is a potential screening strategy of interest in the US. Several studies in the US have examined acceptability of self-sampling for HPV testing and found it acceptable [13–16]. One study among Kentucky Appalachian women found a 100% acceptance rate for clinic-based delivery and return of self-sampling for HPV testing [17]. Another study examined the feasibility and acceptability of a mail-based delivery and return of HPV self-sample kits among low-income women and found a high acceptability rate of 82% for mail-based delivery and return [13]. Few studies, however, have examined women’s preferences for self-sampling for HPV testing considering delivery and return of HPV self-sampling kits and delivery of results. One study found that most women reported to prefer receiving the HPV self-sample kit in the mail (compared to receiving it in the pharmacy or clinic) [13]. This study, however, did not examine possible ways women would prefer to return an HPV self-sample kit (mail, pharmacy drop-off, or clinic drop-off) or how they would prefer to receive HPV test results (mail, phone call, or text message).

The purpose of the current pilot survey study was to assess the relative importance of HPV self-sampling characteristics and willingness to use HPV self-sampling in a specific, but relevant demographic group, Black women from the state of Indiana, U.S.A. The testing characteristics or dimensions included delivery of an HPV self-sampling kit (mail, pharmacy pick-up, clinic pick-up), return of the HPV self-sampling kit (mail, pharmacy drop-off, clinic drop-off), and how women would like the results delivered (mail, phone call, or text message). Ratings-based conjoint analysis (RBCA), a methodological and analytic technique, is uniquely suited to this research focus, as it evaluates how a product’s characteristics (in this case, HPV self-testing) influence product preferences. A secondary aim was to explore sociodemographic characteristics that could be related to self-sampling for HPV testing acceptability. Although studies have begun to examine acceptability related to self-sampling for HPV testing in the US, it is important to understand these preferences along multiple dimensions (delivery and return of HPV self-sampling kits and HPV results delivery) especially among Black women who have higher incidence and mortality from cervical cancer. Results of this pilot survey study could inform future interventions or implementation of self-sampling for HPV testing by determining which factors are most important to women’s willingness to use this technology.

## Methods

### Sample and study design

This study was submitted to and approved as exempt research by the Indiana University IRB. Participants gave verbal consent as signed consent is not required for exempt research according to Indiana University IRB. The participants were provided a verbal and written description of the study. The written description included the following information: that subjects were being asked to participate in research; a description of the study procedures; a statement regarding any potential risks or benefits of participation; a statement that participation is voluntary; and the name, affiliation, and contact information of the researchers.

The data were collected from women who attended the health fair portion of the Annual Indiana Black Expo, a large cultural event that draws an estimated 40,000 attendees. Eligibility inclusion criteria included women who were 1.) Black, 2.) between the ages of 21–65 (age range for cervical cancer screening according to USPSTF guidelines), and 3.) could read and write English. Exclusion criteria included women with a hysterectomy. A sample of 98 women were included in analysis. The sample size was limited by the inclusion criteria, the availability of research personnel, and the many health vendors with displays at the health fair (i.e., many potential participants may have not wanted to take the extra time required to complete the survey). Participants were given the choice between a computer survey administered on REDCap (*n* = 58) or paper surveys (*n* = 40) after determining eligibility and explanation of the study. Participants were compensated with a $20 gift card after completing the survey.

### Measures

Basic sociodemographic characteristics were assessed by self-report, including income (divided at the $30,000 point, which represents approximately 200% of the poverty level for a 2-person household), education, and marital status of participants. Then, participants were given a description of self-sampling for HPV testing and asked to evaluate hypothetical self-sampling scenarios that varied along 3 dimensions: delivery of an HPV self-sampling kit (mail, pharmacy pick-up, clinic pick-up), return of the HPV self-sampling kit (mail, pharmacy drop-off, clinic drop-off), and HPV result delivery (mail, phone call, or text message). A full factorial design would have required women to rate 27 scenarios, which would have imposed undue burden on the respondents. Therefore, we generated a fractional factorial design using the conjoint analysis procedure available in SPSS version 24 (IBM Corp., Armonk, NY) to create nine representative, independent scenarios that allowed us to evaluate the main effects of the dimensions. An example of a scenario presented to participants was “An HPV self-sampling kit delivered by mail that could be returned by mail with results delivered by text message. If self-sampling were available today, and you had time, how likely would you be to complete self-sampling in the given scenario?”. Participants rated the scenarios on an 11-point scale in intervals of 10 points (1–100) where 0 represented that they would never complete self-sampling for HPV testing and 100 meant that they would definitely complete self-sampling for HPV testing. Acceptability for HPV self-sampling in general was evaluated by creating a scale score based on the mean value across the 9 items illustrating hypothetical self-sampling scenarios (Cronbach’s alpha = .94).

### Analysis

We used SPSS 24 to describe sociodemographic characteristics for all 98 participants and the 63 participants who did not assign the same ratings to all scenarios. Then, RBCA was used to examine how HPV self-sample kit characteristics influenced ratings for participants (*n* = 63) who did not assign the same ratings to all scenarios. Participants who assigned the same ratings to all scenarios were not included in analyses. Nine hypothetical self-sampling scenarios were each defined along 3 dimensions: delivery of HPV self-sampling kit (mail, pharmacy pick-up, or clinic pick-up), return of HPV self-sampling kit (mail, pharmacy drop-off, or clinic drop-off), and delivery of HPV results (mail, phone call, text message). Income, education, and marital status were regressed on the HPV testing acceptability measure (created from the 9 items illustrating different hypothetical self-sampling for HPV testing scenarios among all 98 women) in a linear regression model.

RBCA is a regression-based technique used to understand how product preferences are influenced by product attributes and has been validated for use in previous health-related research [18–20]. RBCA allows respondents to consider attributes simultaneously so that respondents can make trade-offs. Conjoint analysis of the 9 scenarios revealed the relative preferences, named part-worth utilities in conjoint analysis, participants placed on each dimension. For example, in the dimension of HPV self-sampling kit return, the preference placed on the attribute of mailed delivery of HPV self-sampling kit is reflected in a higher part-worth utility score compared to the part-worth utility scores of clinic or pharmacy pick-up. A negative part-worth utility score indicates a relative dislike for an attribute (such as clinic pick-up of HPV self-sampling kit) and a positive part-worth utility score shows a relative preference for an attribute. A wider range of part-worth utility attribute scores across a given dimension has a greater influence on importance scores than a dimension with a smaller range in values. The sum of the part-worth utilities needs to equal zero in each of the 3 dimensions. Importance scores were calculated by the relative ranges of part-worth utilities across the 3 dimensions, and in this approach, the sum of importance scores across dimensions must equal 100. The higher the importance score for a given dimension (such as HPV self-sampling kit return), the greater the influence on acceptability of a given scenario.

## Results

### Sample

Sociodemographics (income, education, marital status) of the women who rated the scenarios differently (*n* = 63) and were therefore included in the conjoint analysis and the total sample (*n* = 98) were examined (Table 1). Of the 98 participants, 36% (*n* = 35) rated all the scenarios the same. Sixty percent (*n* = 21) of the sample who rated the scenarios the same held strongly positive views of self-sampling (Table 2) while 23% (*n* = 8) had strongly negative views. Seventeen percent (*n* = 6) held midpoint views. The 35 participants who demonstrated no variability across the vaccine scenarios were necessarily eliminated from the conjoint analysis. The overall conjoint analysis was based on the 63 participants who did not rate all the vaccine scenarios the same and provided responses for all nine scenarios.

**Table 1:** Sociodemographics of those who were included in the conjoint analysis (*n* = 63) and the total sample (*n* = 98)

Variable	Participants included in conjoint analysis (*n* = 63)	Total participants (*n* = 98)
Income, n (%)		
≥ $30,000	17 (27)	34 (35)
< $30,000	45 (73)	63 (65)
Education, n (%)		
< 4-year degree	33 (52)	55 (56)
≥ 4-year degree	30 (48)	43 (44)
Marital Status, n (%)		
Married or partner	24 (38)	34 (35)
Divorced, widowed separated, or single	39 (62)	64 (65)

**Table 2:** Ratings and number of participants who rated all self-sampling for HPV testing scenarios with the same rating (*n* = 35)

Rating	Number of participants
100	21
50	5
25	1
5	1
1	3
0	4

### Self-sampling for HPV testing acceptability

Across the 9 scenarios, overall willingness to self-sample ranged from 0 to 100 with a mean of 60.9 (SD = 31.3). The least acceptable self-sample scenario, “An HPV self-sampling kit picked up at the clinic that could be returned by mail with results delivered by mail”, received a mean score of 60.4 (SD = 36.5). The most acceptable self-sample scenario was “An HPV self-sampling kit picked up at the clinic, returned to the pharmacy, and results delivered by phone” (mean 64.7, SD = 35). Table 3 lists the mean scores and standard deviations of each scenario. A linear regression analysis of income, education, and marital status on the HPV testing acceptability measure (among all 98 women) was not associated with self-sampling acceptability. The overall regression (F = .443, df = 3, *p* = .723) found an R^2^ of .014.

**Table 3:** Self-sampling for HPV testing scenarios presented to participants

HPV self-sampling kit delivery	HPV self-sampling kit return	HPV test result delivery method	Mean Score	Standard Deviation
Mail	Mail	Text Message	63.48	36.82
Mail	Pharmacy	Mail	63.31	36
Mail	Clinic	Phone Call	63.24	35.38
Pharmacy	Clinic	Text Message	62.63	35.13
Pharmacy	Mail	Mail	63.24	35.69
Pharmacy	Pharmacy	Phone Call	63.44	34.61
Clinic	Pharmacy	Phone Call	64.73	34.98
Clinic	Clinic	Text Message	60.51	36.09
Clinic	Mail	Mail	60.35	36.45

**Fig. 1:**
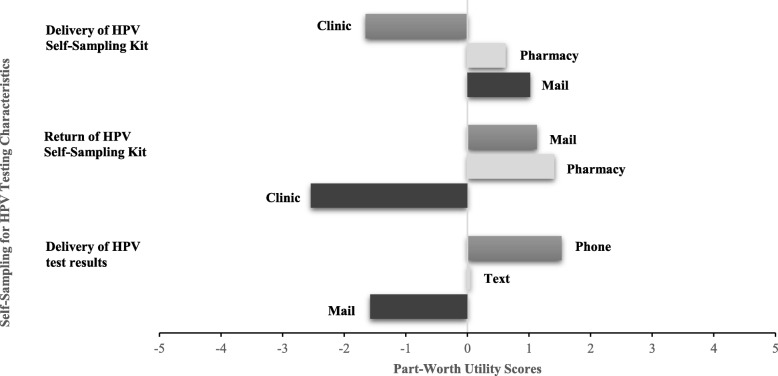
Conjoint analysis of the relative preferences (part-worth utilities) for different self-sampling for HPV testing scenarios

**Fig. 2:**
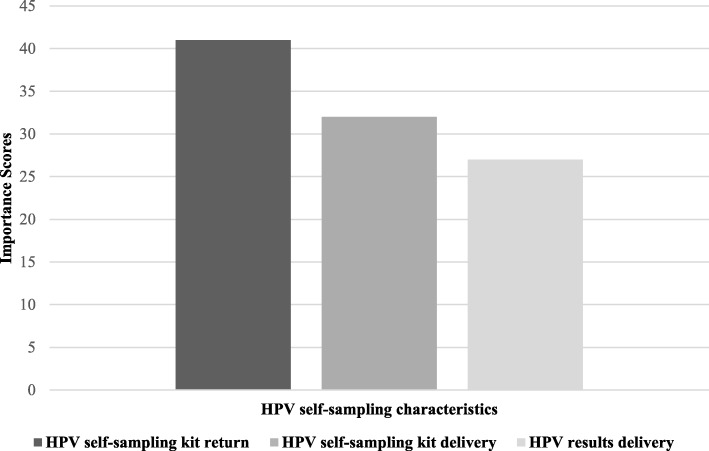
Contribution of each self-sampling for HPV testing characteristic to overall self-sampling importance scores

For the 63 participants in the conjoint analysis, the part-worth utilities in Fig. 1 illustrate that the most important decisional factor (importance score = 40.6 (Fig. 2)) was HPV self-sampling kit return with a preference for returning the HPV self-sampling kit to the pharmacy rather than to the clinic or mailing the HPV self-sampling kit. The next most important decisional factor (importance score = 31.9 (Fig. 2)) was HPV self-sampling kit delivery, and participants preferred mailed delivery of the HPV self-sampling kit over pharmacy or clinic pick-up as illustrated by the part-worth utilities in Fig. 1. The least important decisional factor was the HPV test results delivery (importance score = 27.4 (Fig. 2)) with participants preferring a phone call over a text message or mailed delivery of HPV results (Fig. 1). The conjoint procedure examines correlative fit between the derived fit and data. The overall conjoint analysis model demonstrated a fit with the data with a Pearson R of .98.

## Discussion

### Self-sampling for HPV testing acceptability

The current study evaluated factors related to the willingness of participants to engage in HPV testing for self-sampling. Previous studies that have examined HPV self-sampling acceptability have not used RBCA to allow examination of multiple dimensions of HPV self-sampling that could be important in implementation of the technology. The factors measured in the current study included: delivery of an HPV self-sampling kit (mail, pharmacy pick-up, clinic pick-up), return of the HPV self-sampling kit (mail, pharmacy drop-off, clinic drop-off), and how women would like the results delivered (mail, a phone call, or a text message). Overall acceptability appeared to be mostly positive with a mean score of 60.9 and the lowest mean score 60.4. A previous study using clinic-based delivery and return of self-sampling found an acceptability rate of 100% [17], however, the authors measured acceptability by participation in the study rather than an acceptability scale or conjoint analysis. Another study found an acceptability rate of 82% with a mail-based delivery and return of HPV self-sampling kits using acceptability measures [13]. Our lower overall acceptability rate may reflect that a different methodology, conjoint analysis, was used to examine acceptability. Additionally, the women in the current study had not actually used the HPV self-sample kit, and women may become more comfortable with self-sampling after using an HPV self-sampling kit.

### Self-sampling for HPV testing characteristics

The current study found that most participants (*n* = 63) did discriminate between the different self-sampling for HPV testing scenarios. The most important self-sampling for HPV testing characteristic was kit return with a preference for return to a pharmacy. Although some aspects of HPV self-sampling kit return have been measured before, studies have not examined if other methods of return (pharmacy or clinic) would be preferred to mailed return. A previous study found that a majority of participants (82%) reported being comfortable sending the kit through the mail [13], but most participants who responded to the survey had returned the HPV self-sampling kit through the mail so were most likely comfortable with mailed return. Little is known about the women who did not return the HPV self-sample kit through the mail, and those women may have more problems with mail-based return of HPV self-sampling kits, due to the reduction of neighborhood mail boxes and the difficulty of traveling to a post office [21] . Further research is needed to determine structural barriers related to mailed return of HPV self-sampling kits such as availability of a mailbox, proximity of a mailbox to home, or other access factors. Not surprisingly, clinic-based return of HPV self-sampling kits was least favorable likely because of structural barriers such as transportation and clinic hours. A pharmacy may be open 24 h and have many locations whereas a clinic is usually only open during business hours during the day and may not be as close to home or work as a pharmacy.

The next most important characteristic related to self-sampling for HPV testing was HPV self-sample kit delivery. In line with a previous study that found 99% of women preferred receiving the HPV self-sampling kit in the mail, women in this study preferred to receive an HPV self-sampling kit in the mail as opposed to the clinic or pharmacy [13]. Mail-based delivery overcomes structural barriers related to the least favorable option, the clinic, and is more convenient than the pharmacy.

The least important characteristic related to self-sampling for HPV testing was the HPV test result delivery. Participants preferred receiving a phone call about HPV results, and a previous study found that 97% of women were comfortable with receiving their HPV results over the phone [13]. This may reflect that women want to ask questions or receive more information about their diagnosis than a mailed letter or text message would provide. Prior literature has shown that women often do not understand the implications of a positive HPV test or what a positive HPV diagnosis means [22, 23]. An HPV diagnosis is often associated with stigma, anxiety, and distress [22, 24, 25]. An interaction with a healthcare provider may alleviate these negative feelings more so than an impersonal contact method such as a mailed letter or text message. One study found that women preferred to receive HPV information from a healthcare provider rather than printed information [26]. Women may also be worried about loss of confidentiality of their diagnosis in a mailed letter or text message. Further research is needed to determine the best methods for delivering HPV results to patients and the HPV information that patients would like to know.

This study has several limitations. First, the participants were all from a minority health fair and Black, which may limit generalizability. Other populations may find self-sampling for HPV testing more or less acceptable and/or have different preferences than this specific subgroup. For example, a study of British women found that HPV testing was more acceptable among White women [27]. The higher educational levels of this sample may not be representative of all Black populations. Second, the sample size of women who did not answer all the scenarios the same was small (*n* = 63). Further research with a larger, more representative population is necessary to more fully understand characteristics related to acceptability of self-sampling for HPV testing and potential sociodemographic, health belief, and knowledge predictors of acceptability. Third, other self-sampling characteristics not measured, such as device type or invitation method, could be more important than those measured in the current study. For example, a recent study found that most participants responded to an invitation to opt into a self-sampling for HPV testing study by mail (61%) or online (37%) rather than by phone (1%) or email (< 1%) [28]. Due to the repetitive nature of the scenarios, participants may have felt response burden to the questions and were more likely to leave a question blank, answer the scenarios with the same score, or did not make trade-offs when considering self-sampling characteristics. Fourth, although a description of self-sampling for HPV testing was provided, participants may have had difficulty imagining the kit and scenarios. This could be potentially overcome by showing participants a kit and instructions for self-sampling prior to administration of a survey.

## Conclusions

In summary, self-sampling was found to be generally acceptable to Black women in this pilot survey study. The most important characteristic among the women related to self-sampling was the return of the HPV self-sampling kit, second, delivery of the kit, and finally the HPV results delivery method. Sociodemographic characteristics including income, education, and marital status were not related to self-sampling for HPV testing acceptability. If these findings can be replicated among the general population or underscreeners, the information could be used by researchers developing interventions related to self-sampling for HPV testing and the implementation of self-sampling among providers.

## Data Availability

The datasets used and analyzed during the current study are available from the corresponding author upon reasonable request.

## Abbreviations

HPV: Human papillomavirus
USPTSF: (United States Preventive Services Task Force)
RBCA: Ratings-based conjoint analysis
